# Supplementary material for: Dynamic Cerebral Autoregulation Post Endovascular Thrombectomy in Acute Ischemic Stroke
Source: Brain Sci. 2020 Sep 16;10(9):641. doi: 10.3390/brainsci10090641 (PMC7564150; doi:10.3390/brainsci10090641)
Supplement: Supplementary file 1 [file brainsci-10-00641-s001.pdf]

brain sciences

MDPI

Brief Report

Dynamic Cerebral Autoregulation Post Endovascular Thrombectomy in Acute Ischemic Stroke - Supplementary

Supplemental Table 1. Coherence, gain, and phase calculated for the VLF (very low frequency) band (0.02–0.03 Hz) and LF(low frequency) band (0.03–0.07 Hz) for subjects and controls at each time-point. Ipsilateral and contralateral hemisphere results are shown.

Coherence.

VLF (0.02–0.03 Hz)	N	Mean	SD	SE	CI	LF (0.03–0.07 Hz)	N	Mean	SD	SE	CI
**<24 h**						**<24 h**					
Ipsilateral	61	0.504	0.248	0.032	0.063	Ipsilateral	61	0.541	0.209	0.027	0.054
Contralateral	59	0.453	0.241	0.031	0.063	Contralateral	59	0.517	0.196	0.026	0.051
**24–72 h**						**24-72 h**					
Ipsilateral	41	0.563	0.262	0.041	0.083	Ipsilateral	41	0.593	0.207	0.032	0.065
Contralateral	41	0.531	0.234	0.037	0.074	Contralateral	41	0.552	0.194	0.030	0.061
**72–96 h**						**72-96 h**					
Ipsilateral	24	0.550	0.244	0.050	0.103	Ipsilateral	24	0.553	0.167	0.034	0.070
Contralateral	24	0.530	0.258	0.053	0.109	Contralateral	24	0.517	0.176	0.036	0.074
**>96 h**						**>96 h**					
Ipsilateral	19	0.454	0.216	0.050	0.104	Ipsilateral	19	0.461	0.167	0.038	0.081
Contralateral	19	0.438	0.225	0.052	0.109	Contralateral	19	0.393	0.174	0.040	0.084

Gain.

VLF (0.02–0.03 Hz)	N	Mean	SD	SE	CI	LF (0.03–0.07 Hz)	N	Mean	SD	SE	CI
**<24 h**						**<24 h**					
Ipsilateral	61	0.526	0.361	0.046	0.092	Ipsilateral	61	0.563	0.374	0.048	0.096
Contralateral	59	0.551	0.364	0.047	0.095	Contralateral	59	0.587	0.308	0.040	0.080
**24-72 h**						**24-72 h**					
Ipsilateral	41	0.554	0.339	0.053	0.107	Ipsilateral	41	0.539	0.287	0.045	0.091
Contralateral	41	0.648	0.324	0.051	0.102	Contralateral	41	0.591	0.249	0.039	0.079
**72-96 h**						**72-96 h**					
Ipsilateral	24	0.696	0.467	0.095	0.197	Ipsilateral	24	0.714	0.284	0.058	0.120
Contralateral	24	0.823	0.567	0.116	0.239	Contralateral	24	0.875	0.451	0.092	0.191
**>96 h**						**>96 h**					
Ipsilateral	19	0.646	0.332	0.076	0.160	Ipsilateral	19	0.668	0.312	0.071	0.150
Contralateral	19	0.670	0.363	0.083	0.175	Contralateral	19	0.631	0.312	0.072	0.150

Phase.

VLF (0.02–0.03 Hz)						LF (0.03–0.07 Hz)					
	N	Mean	SD	SE	CI		N	Mean	SD	SE	CI
**<24 h**						**<24 h**					
Ipsilateral	61	0.581	0.723	0.093	0.185	Ipsilateral	61	0.684	0.498	0.064	0.127
Contralateral	59	0.649	0.911	0.119	0.237	Contralateral	59	0.918	0.498	0.065	0.130
**24–72 h**						**24–72 h**					
Ipsilateral	41	0.452	0.881	0.138	0.278	Ipsilateral	41	0.689	0.552	0.086	0.174
Contralateral	41	0.565	0.848	0.132	0.268	Contralateral	41	0.860	0.471	0.074	0.149
**72–96 h**						**72–96 h**					
Ipsilateral	24	0.440	0.898	0.183	0.379	Ipsilateral	24	0.648	0.563	0.115	0.238
Contralateral	24	0.513	0.811	0.166	0.343	Contralateral	24	0.713	0.687	0.140	0.290
**>96 h**						**>96 h**					
Ipsilateral	19	0.713	0.989	0.227	0.476	Ipsilateral	19	0.704	0.606	0.139	0.292
Contralateral	19	0.525	1.027	0.236	0.495	Contralateral	19	0.668	0.644	0.148	0.311
